# Extraction, Preliminary Characterization and Evaluation of *in Vitro* Antitumor and Antioxidant Activities of Polysaccharides from *Mentha piperita*

**DOI:** 10.3390/ijms150916302

**Published:** 2014-09-15

**Authors:** Xin Liu, Zhen-Liang Sun, Ai-Rong Jia, Ya-Ping Shi, Rui-Hong Li, Pei-Ming Yang

**Affiliations:** 1 Biology Institute of Shandong Academy of Sciences/Key Laboratory for Applied Microbiology of Shandong Province, Jinan 250014, China; E-Mails: rock0515@163.com (X.L.); jiaar@sdas.org (A.-R.J.); hand-user@163.com (Y.-P.S.); 2 State Key Laboratory of New Drug & Pharmaceutical Process, Shanghai Institute of Pharmaceutical Industry, Shanghai 200040, China; E-Mail: hope1126@hotmail.com; 3 Fengxian Hospital Affiliated to Southern Medical University, 6600 NanFeng Road, Shanghai 201499, China

**Keywords:** *Mentha piperita*, response surface methodology, polysaccharide, antitumor, antioxidant

## Abstract

This study describes the extraction, preliminary characterization and evaluation of the* in vitro* antitumor and antioxidant activities of polysaccharides extracted from *Mentha piperita* (MPP). The optimal parameters for the extraction of MPP were obtained by Box-Behnken experimental design and response surface methodology (RSM) at the ratio of water to raw material of 20, extraction time of 1.5 h and extraction temperature at 80 °C. Chemical composition analysis showed that MPP was mainly composed of glucuronic acid, galacturonic acid, glucose, galactose and arabinose, and the molecular weight of its two major fractions were estimated to be about 2.843 and 1.139 kDa, respectively. *In vitro* bioactivity experiments showed that MPP not only inhibited the growth of A549 cells but possessed potent inhibitory action against DNA topoisomerase I (topo I), and an appreciative antioxidant action as well. These results indicate that MPP may be useful for developing safe natural health products.

## 1. Introduction

Cancer is a leading cause of death worldwide and a diverse group of diseases characterized by the uncontrolled proliferation of anaplastic cells, leading to invasion of surrounding tissues and metastasis to other organs [1]. It is one of the most lethal diseases severely threatening to human life. Chemotherapy is one of the most frequently used therapeutic modalities for the treatment of cancer, but the therapeutic outcome is usually unsatisfactory due to serious and intolerable adverse side effects. Therefore, it is very important to develop novel antitumor substances with little toxicity to the host. In recent decades, numerous polysaccharides isolated from natural materials have been proven to have few adverse effects and possess a wide range of biological functions such as antitumor and antioxidant properties [2,3,4,5]. Natural polysaccharides may prove to be important potential pharmaceuticals for the prevention and treatment of cancer in the future.

*Mentha piperita* L., belonging to the family *Lamiaceae*, is a perennial herb native to Europe, sparingly naturalized in the northern USA and Canada, and cultivated in many parts of the world. It used to be known as a hybrid of *M. spicata* L. (spearmint) and *M. aquatic* (water mint) [6]. It is best known for its fragrance and flavor compositions. In Eastern and Western traditional medicines, peppermint and its oil have been used in antispasmodics, aromatics, antiseptics or even medications for the treatment of colds, cramps, indigestions, nausea, sore throat, toothache or even cancer [7]. Modern pharmacology research has demonstrated that *M. piperita* possesses antioxidant, antitumor, antiallergenic, antiviral and antibacterial activities [8,9,10,11,12]. So far, research on *M. piperita* has focused on peppermint oil and small molecules, and there is no published information about the biological and physiochemical properties of the polysaccharides extracted from *M. piperita*.

Polysaccharides are polymeric carbohydrate structures, formed of repeating units joined together by glycosidic bonds. Aqueous extraction is the most common method of extraction of plant polysaccharides, and the extraction process is usually optimized by mathematics. Response surface methodology (RSM) is an empirical statistical technique for multiple regression analysis by using quantitative data obtained from properly designed experiments to solve multivariate equations simultaneously. This methodology consists of a group of mathematical and statistical procedures that can be used to evaluate the functional form of a process, involving one or more dependent variables influenced by various factors or independent variables. This methodology has been widely used to optimize the polysaccharides extraction process [13,14,15,16]. Knowing that RSM can simplify the complexity of experimental trials needed to evaluate multiple variables and their interactions, the objective of this study was to optimize the extraction process of polysaccharides from *M. piperita* using RSM, and to investigate their antitumor and antioxidant activities* in vitro*.

## 2. Results and Discussion

### 2.1. Statistical Analysis and Model Fitting

On the basis of preliminary experiments (data not shown), the three parameters: ratio of water to raw material (*A*), extraction time (*B*) and extraction temperature (*C*) were selected as the variables to optimize the extraction conditions of polysaccharide from *M. Piperita* (MPP). A 17-run Box-Behnken design (BBD) was applied statistically to optimize *Y* (MPP yield) under different experimental combinations and is presented in Table 1. There was a considerable variation of *Y* depending on the different extraction conditions. MPP yields ranged from 5.140% to 8.452%.

**Table 1:** Box-Behnken Design (BBD) experimental design with the independent variables.

Run	*A*	*B* (h)	*C* (°C)	*Y* MPP Yield (%)
1	15	1.0	80	5.140
2	25	1.0	80	7.376
3	15	2.0	80	5.769
4	25	2.0	80	6.588
5	15	1.5	70	5.592
6	25	1.5	70	7.162
7	15	1.5	90	5.838
8	25	1.5	90	7.463
9	20	1.0	70	7.486
10	20	2.0	70	7.058
11	20	1.0	90	7.152
12	20	2.0	90	7.324
13	20	1.5	80	7.989
14	20	1.5	80	8.452
15	20	1.5	80	8.328
16	20	1.5	80	8.021
17	20	1.5	80	8.371

Using multiple regression analysis on the experimental data, the response variable and the test variables were related by the following second-order polynomial equation:

*Y* = 82.32 + 7.81*A* − 0.52*B* + 0.60*C* − 3.54*AB* + 0.14*AC* + 1.50*BC* − 13.78*A*^2^ − 6.36*B*^2^ − 3.41*C*^2^ (1)

The coefficient of determination (*R*^2^ = 0.9862), the adjusted coefficient of determination (*R*^2^_Adj_ = 0.9684) and the coefficient of variation (C.V. = 2.55%) are shown in Table 2, where the values indicate that the accuracy and the general availability of the polynomial model are adequate, and the *R*^2^_Pred_ of 0.9345 is in reasonable agreement with the *R*^2^_Adj_. The “Adequate Precision” of 22.21 indicated that this model could be used to navigate the design space.

The significance of each coefficient was checked using the *F*-test and the *p*-value (Table 2). The Model *F*-value of 55.48 implied that the model was significant, and there was only a 0.01% chance that a “Model *F*-value” that was this large occurred because of noise. The “Lack of Fit *F*-value” of 0.38 implied that the Lack of Fit was not significant relative to the pure error, and there was a 22.27% chance that a “Lack of Fit *F*-value” of such a size could have occurred because of noise. As shown in Table 2, the linear coefficients (*A*), a quadratic term coefficient (*A*^2^, *B*^2^, *C*^2^) and cross product coefficients (*AB*) were significant, with very small *p*-values (*p* < 0.05). The other term coefficients were not significant (*p* > 0.05). Therefore, *A*, *A*^2^, *B*^2^, *C*^2^ and* AB* were important factors in the extraction process of the polysaccharides.

**Table 2:** Analysis of variance (ANOVA) of the experimental results of the BBD.

Source	Sum of Squares		Degree of Freedom	Mean Square		*F*-Value		*p*-Value
Model	16.51		9	1.83		55.48		<0.0001
*A*	4.88		1	4.88		147.70		<0.0001
*B*	0.022		1	0.022		0.65		0.4462
*C*	0.029		1	0.029		0.87		0.3826
*AB*	0.50		1	0.50		15.18		0.0059
*AC*	7.563 × 10^−^^4^		1	7.563 × 10^−4^		0.023		0.8840
*BC*	0.090		1	0.09		2.72		0.1492
*A*^2^	7.99		1	7.99		241.71		<0.0001
*B*^2^	1.71		1	1.71		51.58		0.0002
*C*^2^	0.49		1	0.49		14.80		0.0063
Residual	0.23		7	0.033		-		-
Lack of Fit	0.051		3	0.017		0.38		0.7761
Pure Error	0.18		4	0.045		-		-
Cor Total	16.74		16	-		-		-
Standard Deviation	Mean	C.V.%	Press	*R*^2^	*R*^2^*_Adj_*		*R*^2^*_Pred_*	Adequate precision
0.18	7.12	2.55	1.10	0.9862	0.9684		0.9345	22.21

### 2.2. Analysis of Response Surface

**Figure 1:**
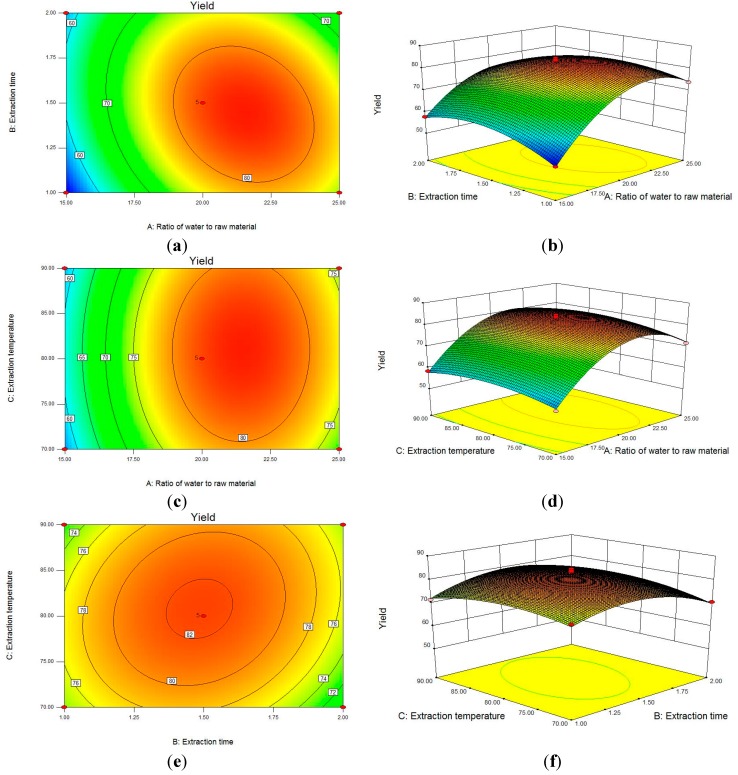
Contour plots (**a**,**c**,**e**) and response surface plots (**b**,**d**,**f**) showing effect of ratio of water to raw material (*A*), extraction time (*B*) and extraction temperature (*C*) on the yield of polysaccharide from *Mentha piperita* (MPP).

The relationship between independent and dependent variables was illustrated in 3D response surfaces and 2D contour plots generated by the model for yield of polysaccharides, and two variables were depicted in one tri-dimensional surface plot while the other variable remained at zero level. Figure 1a,b show that the effect of the ratio of water to raw material (*A*), extraction time (*B*) and their reciprocal interaction on polysaccharide yield, when the extraction temperature (*C*) was fixed at zero level. *A* and *B* were shown to have a quadratic effect on the yield. When *A* was kept at a lower level, the yield increased first and then decreased with the increase of *B*, and the mutual interaction between *A* and *B* was significant. Likewise, Figure 1c,d show that the ratio of water to raw material (*A*) and extraction temperature (*C*) had a quadratic effect on the yield when extraction time (*B*) was fixed at 1.5 h. As shown in Figure 1e,f, when the ratio of water to raw material (*A*) was fixed at 20, the polysaccharide yield was insignificant with increase of the extraction time (*B*). The extraction temperature (*C*) was shown to have a quadratic effect on the response.

Analysis of the plots showed that the optimal conditions for MPP extraction were the ratio of water to raw material at 21.49, the extraction time of 1.44 h, and the extraction temperature at 80.69 °C. Under these optimal conditions, the maximum predicted yield to MPP was 8.354%, and the experimental yield was 8.281% ± 0.387%, which agreed with the predicted value. In view of practicality of operation, the extraction process is better when the ratio of water to material is at 20, an extraction time of 1.5 h and an extraction temperature at 80 °C, respectively. Therefore, the results indicated the suitability of the model employed and the success of RSM in optimizing the extraction conditions for MPP.

### 2.3. Physicochemical Properties of MPP (Mentha piperita)

**Figure 2:**
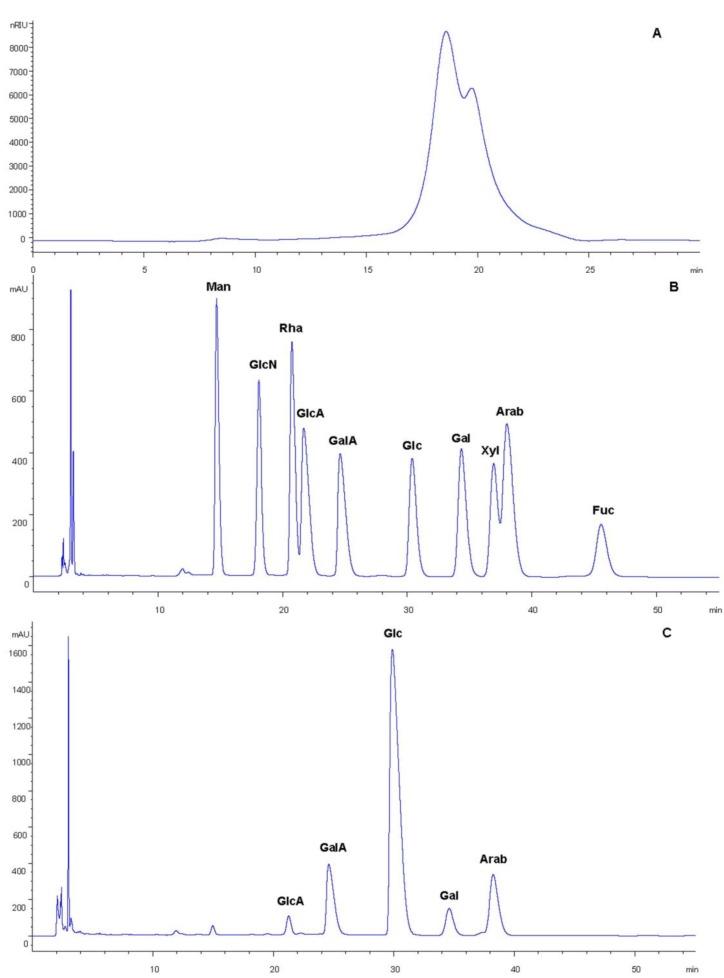
High performance gel permeation chromatography (HPGPC) chromatogram of MPP on TSKgel G3000 PW_XL_ column (Tosoh Corporation, Yamaguchi, Japan) (**A**); Reversed-phase high performance liquid chromatography (HPLC) analysis of monosaccharide composition in MPP. The standards were separated under the conditions described in (**B**); and the derivatives of MPP hydrolysate were separated described in (**C**) (Man, mannose; GlcN, glucosamine; GlcA, glucuronic acid; Rha, rhamnose; GalA, galacturonic acid; Glc, glucose; Gal, galactose; Xyl, xylose; Arab, arabinose; Fuc, fucose).

The chemical composition analysis indicated that the obtained MPP contained 70.48% ± 1.75% sugar, 14.70% ± 0.48% protein, 3.89% ± 0.16% sulfate and 8.96% ± 1.33% uronic acid. Moreover, MPP was heterogeneous and contained two major fractions, the molecular weight of which was estimated to be about 2.843 and 1.139 kDa, respectively (Figure 2A). The results of the monosaccharide composition analysis of MPP are shown in Figure 2B,C. HPLC (high performance liquid chromatography) analysis showed that MPP mainly consisted of glucuronic acid, galacturonic acid, glucose, galactose and arabinose.

**Figure 3:**
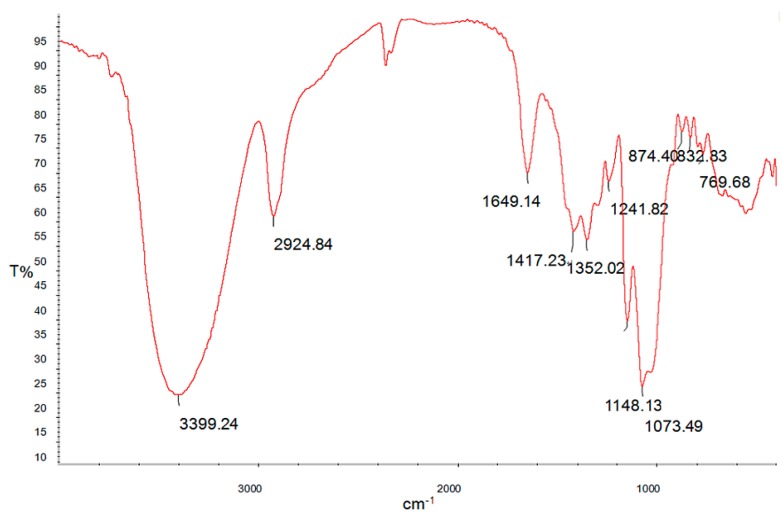
Fourier Transform Infrared (FT-IR) spectroscopy of MPP.

FT-IR (Fourier Transform Infrared) spectroscopy is a powerful tool for identification of characteristic organic groups in the polysaccharides. As shown in Figure 3, there are two characteristic absorptions bands of polysaccharides: a strong and wide absorption band at about 3399 cm^−1^ for O-H stretching vibration, and a band in the region of 2924 cm^−1^ for C-H stretching vibration. The band at about 1073 cm^−1^ was assigned to the valent vibrations of the C-O-C bond and glycosidic bridge. The characteristic absorptions at 874 and 832 cm^−1^ indicate that α- and β-configurations of the sugar units might simultaneously exist in MPP. These results provide valuable information for further structural study on MPP.

### 2.4. Antitumor Activity in Vitro

#### 2.4.1.* In* *Vitro* Cancer Cell Line Cytotoxicity Assay

**Figure 4:**
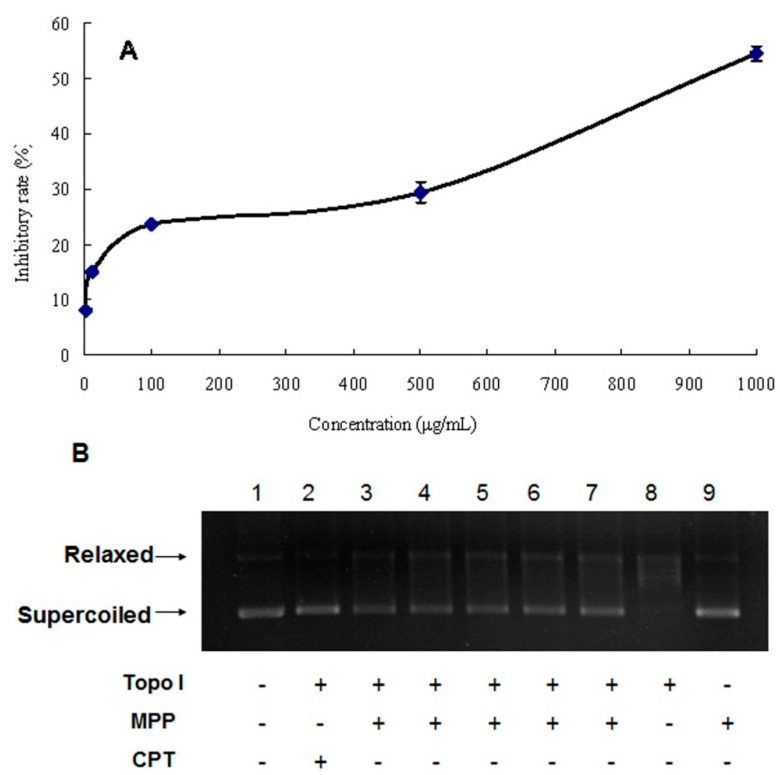
Antitumor activity of MPP, (**A**) Inhibitory effect of MPP on A549 cell lines; (**B**) Effect of MPP on the relaxation activity of topoisomerase I (Topo I). Lane 1, DNA alone (no topo I and no MPP); Lane 2, camptothecin (CPT) at concentration of 50 μg/mL + DNA + topo I; Lane 3–7, MPP at concentrations of 25, 50, 100, 200 and 400 μg/mL + DNA + topo I, respectively; Lane 8, topo I + DNA; Lane 9, DNA + 200 μg/mL MPP.

To explore the biological activity of MPP, the inhibitory effect on A549 non-small cell lung adenocarcinoma cells was investigated using 3-(4,5-dimethylthiazol-2-yl)-2,5-diphenyltetrazolium bromide (MTT) method (Figure 4A). The results showed that MPP had a moderate toxic effect on A549 cell line, with IC_50_ value of 879.52 ± 22.55 μg/mL. The growth of A549 cells was inhibited by MPP in a dose-dependent manner. The inhibitory rate was 54.54% ± 1.38% at the highest concentration tested (1 mg/mL).

#### 2.4.2. DNA Topoisomerase I Inhibitory Activity

The inhibitory activity of MPP against topo I was evaluated by agarose-gel electrophoresis. As shown in Figure 4B, lane 1 is DNA alone; lane 8 is topo I together with DNA, and supercoiled DNA was relaxed by topo I completely; in lane 9, the system consisted of DNA and 200 μg/mL MPP. Lane 1 was similar to lane 9, indicating that MPP could not combine with DNA. In lanes 3–7, MPP, DNA and topo I existed in the same system. The results showed that MPP was the inhibitor of topo I that can inhibit the relaxation activity of topo I toward DNA, with IC_50_ value of 340.81 μg/mL. It was also found that the inhibitory activity of MPP weakened gradually with decreasing concentration.

DNA topo I is the ubiquitous enzyme that can regulate DNA topological structures by sequential breakage and reunion of DNA single strand. It was reported to be involved in DNA transcription, replication and recombination [17]. DNA topo I inhibitory effect has become an important target for cancer treatment, and some inhibitors against DNA topo I have been used in clinical treatment. Although many natural polysaccharides are proposed to have antitumor activities, few studies have reported polysaccharides that can inhibit DNA topo I activity. Japanese researchers reported a polysaccharide derived from marine microalgae that possessed notable topo I inhibitory activity, and their finding may promote further study on MPP [18]. According to the previous study, polysaccharides with high molecular weight could hardly be incorporated into the nucleus and directly inhibit topo I, and therefore it is reasonable to assume that the low molecular weight polysaccharides existing in MPP might be the active substances [18]. Further investigation is necessary to investigate the structure and action mechanism of MPP.

### 2.5. In Vitro Antioxidant Activities of MPP

#### 2.5.1. DPPH (1,1-Diphenyl-2-picryl-hydrazyl0) Radical Scavenging Assay

**Figure 5:**
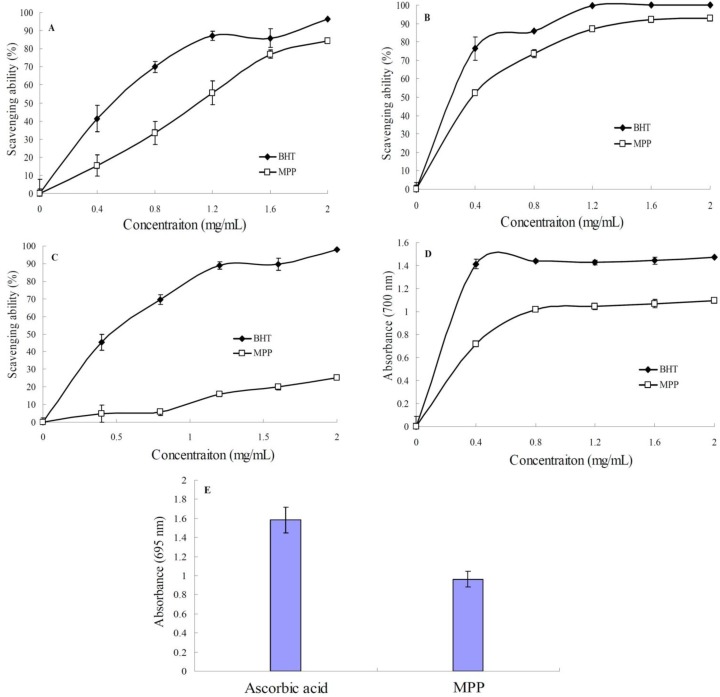
Antioxidant activities of MPP, (**A**) scavenging of 1,1-diphenyl-2-picryl-hydrazyl (DPPH) radical; (**B**) scavenging of hydroxyl radical; (**C**) scavenging of superoxide radical; (**D**) reducing power and (**E**) total antioxidant activity. Values are representative of three separate experiments.

DPPH (1,1-Diphenyl-2-picryl-hydrazyl) is a stable free radical and has been widely accepted as a method for evaluating free radical scavenging activities of antioxidants [19]. MPP clearly displayed scavenging activity against DPPH in a concentration-dependent manner (Figure 5A). The effect of MPP on scavenging DPPH was 15.43% at 0.4 mg/mL, and the scavenging rate increased to 84.48% at 2 mg/mL. The scavenging ability of MPP (IC_50_ = 1.13 mg/mL) was similar to that of butylated hydroxytoluene (BHT) (IC_50_ = 0.71 mg/mL), suggesting that MPP could be used as a free radical inhibitor and primary antioxidant.

#### 2.5.2. Hydroxyl Radical Scavenging Activity

Among the oxygen radicals, hydroxyl radical is the most reactive and can induce severe damage to adjacent biomolecules [19]. As shown in Figure 5B, both MPP and BHT exhibited hydroxyl radical scavenging activity. Within the concentration range of 0.4–2.0 mg/mL, the hydroxyl radical scavenging ability of MPP increased firstly with increasing concentration, and then became gradually inconspicuous when the concentration was beyond 1.2 mg/mL, indicating that MPP has good hydroxyl scavenging activity and can thus be used as a good hydroxyl radical scavenger.

#### 2.5.3. Superoxide Radical Scavenging Activity

Superoxide radical is a reduced form of molecular oxygen created by receiving one electron from mitochondrial electron transport systems. The superoxide radical plays an important role in formation of hydrogen peroxide, hydroxyl radical, or singlet oxygen, which induces oxidative damage in lipids, proteins and DNA. It is a highly toxic species generated by numerous biological and photochemical reactions [4]. The scavenging effect of MPP on superoxide radical is shown in Figure 5C. The superoxide scavenging rate increased from 4.78%–25.17%, when the concentration increased from 0.4–2.0 mg/mL, showing a concentration-dependent manner. Although the scavenging ability of MPP increased with increase of the concentration, its scavenging ability was lower than that of BHT, indicating that MPP had weak superoxide radical scavenging ability.

#### 2.5.4. Reducing Power

The reduction of Fe ions is often used as an indicator of electron-donating activity. Figure 5D showed that the reducing power of MPP increased to a certain extant and then leveled off within the concentration range of 0.4–2.0 mg/mL. Although the reducing power of MPP was lower than that of BHT, it still reached 1.02 at the concentration of 0.8 mg/mL, indicating that MPP has a moderate reducing power. The result suggests that MPP may function as a good electron and hydrogen donor, and therefore should be able to terminate radical chain reactions by converting free radicals to more stable products.

#### 2.5.5. Total Antioxidant Activity

Total antioxidant activity assay is based on the reduction of Mo (VI) to Mo (V) and subsequent formation of a green phosphate/Mo (V) complex in acid medium. As shown in Figure 5E, total antioxidant capacity of MPP was expressed as the number of equivalents of ascorbic acid. MPP exhibited effective antioxidant activity, which is equivalent to 60% of the antioxidant activity of ascorbic acid.

It is common knowledge that oxidation is imperative to many organisms for energy production. However, uncontrolled production of oxygen-derived free radicals can damage cellular components such as lipids and DNA, which may bring about diseases such as cancer, rheumatoid arthritis and atherosclerosis [20]. It has been reported that natural polysaccharides from plants possess antioxidant activities. The* in vitro* antioxidant activity test of the present study showed that MPP had rational DPPH, hydroxyl and superoxide radical scavenging activities, a moderate reducing power and total antioxidant activity. According to previous studies, antioxidant activity of polysaccharide depends on several structural parameters such as the degree of sulfation, the molecular weight, the sulfation position, type of monosaccharide and glycosidic branching [21]. In this study, MPP possessed low molecular weight and high content of glucose, and this special characterization and composition might be propitious to the formation of hydrogen bonds and enhance the hydrogen atom donating ability of MPP to scavenge free radicals, which might improve its antioxidant activity. On the other hand, the level of anti-oxidation and reactive oxygen species correlates well with the generation and malignant transformation of cancer cells. If substances can enhance the level of anti-oxidation and clear the reactive oxygen species in cancer cells, they may inhibit cell growth [2]. This suggests that MPP has antioxidant activity and can protect the organs against damage from free radicals, or retard the progression of disease. Therefore, the antitumor activity of MPP might be attributed to its potential antioxidant activity.

An efficient extraction process was employed to extract MPP from *M. piperita* and the corresponding extraction parameters were optimized by RSM. Although MPP has a high sugar content, it is still a mixture including protein, uronic acid and other impurities. These components might partially affect the antitumor and antioxidant activities of MPP. Meanwhile, molecular weight distribution analysis also showed that MPP was heterogeneous and contained two major fractions, and the relationship between its structural characteristics and biological activities is unknown. Therefore, it is necessary to carry out further studies on the precise chemical structures and biological function mechanism of MPP.

## 3. Experimental Section

### 3.1. Experiment Materials and Chemicals

Stems and leaves of* M. piperita* were collected from the Experimental Halophytes Growing Base of Shandong Academic of Sciences (Jinan, China) in August 2012, dried at room temperature and ground into powder. After passing through a 10 mesh sieve, the powder was stored in the refrigerator. Fetal bovine serum (FBS) and RPMI1640 medium were purchased from Gibco Industries (Grand Island, NY, USA). A549 non-small cell lung adenocarcinoma cell line was purchased from Shanghai Institute of Cell Biology, Chinese Academy of Sciences (Shanghai, China). Topoisomerase I (calf thymus), buffer, bovine serum albumin (BSA), loading buffer and supercoiled pBR322 DNA were all from TaKaRa Biotechnology Co., Ltd. (Dalian, China). Butylated hydroxytoluene (BHT), 3-(4,5-dimethylthiazol-2-yl)-2,5-diphenyltetrazolium bromide (MTT), camptothecin (CPT), 1,1-diphenyl-2-picryl-hydrazyl (DPPH), standard monosaccharides and ascorbic acid were obtained from Sigma-Aldrich (St. Louis, MO, USA). Dextran T-series standards (*M*_W_ (molecular weight): 180, 2500, 4600, 7100, 10,000, 21,100 and 47,100 Da) were from the National Institute for Drugs and Biological Products (Beijing, China). Other reagents used in this study were of the highest quality available from commercial vendors.

### 3.2. Preparation of Polysaccharides

Dried* M. piperita* stems and leaves (1000.0 g) were ground in a rotary mill and sieved (10 mesh) to obtain a fine powder as pretreated samples. They were then extracted with 90% ethanol at 80 °C for 2 h × 3 to defat and remove colored materials and fat-soluble small molecule materials. The pretreated dried samples were extracted with distilled water, with the temperature of water bath kept at a constant temperature within ±1.0 °C, and an electric mixing paddle was used for a given time during the entire extraction process. The extracted slurry was centrifuged at 4000× *g* for 10 min to collect the supernatant, and the insoluble residue was treated once again as mentioned above.

The supernatant was incorporated and concentrated to one-fifth of the initial volume using a rotary evaporator (RE-52 99, Yarong Technology and Science Inc., Shanghai, China) at 60 °C under vacuum. The resulting solution was mixed with four volumes of dehydrated ethanol (ethanol final concentration, 80%) and kept at 4 °C overnight. Then the solution was centrifuged at 4000×* g* for 15 min, washed three times with dehydrated ethanol and dried under reduced pressure. The crude polysaccharides obtained, named MPP, was weighed with a balance (BS2202, SARTOUIS, Göttingen, Germany), and the polysaccharide extraction yield (%) was calculated as follows: polysaccharide extraction yield (%, *w*/*w*) = [dried crude polysaccharide weight (g)/powder weight (g)] × 100%.

### 3.3. Experimental Design

A 17-run Box-Behnken design (BBD) was applied statistically to optimize polysaccharide extraction from *M. piperita*. Knowing that the ratio of water to raw material, extraction time and extraction temperature significantly influenced the yield of polysaccharides, they were designated as *X*_1_ (*A*), * X*_2_ (*B*) and* X*_3_ (*C*) in Table 1, and prescribed into three levels, coded +1, 0 and −1 for high, intermediate and low values, respectively. To minimize the effect of unexplained variability in the observed responses due to systematic errors, all the experiments were carried out at random. The variables were coded according to the following equation:

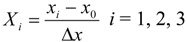
(2)


In this equation *X_i_* is the coded value of the independent variable,* x_i_* is the actual value, *x*_0_ is the value of *x_i_* at the center point, and Δ*x* is the step change. To predict the optimized conditions, a second-order polynomial model was fitted to correlate the relationship between the independent variables and the response (polysaccharide yield).

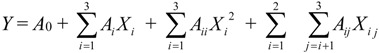
(3)
where *Y* is the dependent variable (the polysaccharide extraction yield), *A*_0_ is an intercept, and *A_i_*, *A_ii_* and *A_ij_* are the coefficients estimated by the model. *X_i_* and *X_j_* are the levels of the independent variables that represent the linear, quadratic and cross-product effects of the *X*_1_, *X*_2_ and *X*_3_ factors on the response, respectively. The model evaluated the effect of each independent variable on the response. The experimental design was analyzed and the predicted data were calculated using the Design-Expert software (v.8.0.6.1 trail, State-East, Inc., Minneapolis, MN, USA) in order to estimate the response of the independent variables. The designed variables in coded units are given in Table 1 along with the experimental values of the response.

### 3.4. Component Analysis

The total sugar content was determined by the phenol-sulfuric acid method using glucose as the standard [22]. The protein content was measured by the method of Bradford [23]. The sulfate ester content was estimated according to previous literature [24]. The uronic acid content was determined by the carbazole-sulfuric acid method [25]. The molecular weight of MPP was determined by high performance gel permeation chromatography (HPGPC) on a HPLC system equipped with a TSKgel G3000 PW_XL_ column calibration (7.8 mm × 30.0 cm) by eluting with 0.2 mol/L Na_2_SO_4_ at 0.5 mL/min The eluate was monitored by a refractive index detector. Column calibration was performed with standard dextrans (*M*_W_: 180, 2500, 4600, 7100, 10,000, 21,100 and 47,100 Da). The calibration curve of Log *M*_W_ (molecular weight) of standard dextrans against their elution time (ET) was obtained (Log *M*_W_ = −0.3151ET + 9.3524, *R*^2^ = 0.9959) [26].

Monosaccharide composition was determined by reversed-phase high performance liquid chromatography (HPLC) after pre-column derivatization and UV detection with minor modifications [26]. Five mg MPP was hydrolyzed with 2 mol/L trifluoroacetic acid at 100 °C for 6 h. Excessive acid was removed by co-distillation with methanol after hydrolysis. One hundred mg dry hydrolysate was dissolved in 100 μL 0.3 mol/L NaOH, and added to 120 μL 0.5 mol/L methanol solution of 1-phenyl-3-methyl-5-pyrazolone (PMP) at 70 °C for 1 h. The mixture was added to 100 μL 0.3 mol/L HCl, vigorously shaken, and then centrifuged at 2400× *g* for 5 min. The supernatant containing the labeled carbohydrate was filtered through 0.22 μm nylon membranes (MSI, Westborough, MA, USA) and 10 μL of the filtrate was injected into the C_18_ column (Kromasil, 4.6 × 250 mm, particle size 5 μm, Akzo Nobel, Sweden). The sample was analyzed by a HPLC system (Aglient 1260 Infinity HPLC, Agilent Technologies, Santa Clara, CA, USA). The mobile phase was a mixture of 0.1 mol/L KH_2_PO_4_ (pH 10) and acetonitrile (87:13). The flow rate was set at 1.0 mL/min and column temperature was set at 30 °C. Sugar identification was done by comparison with reference sugars (mannose, glucosamine, glucuronic acid, rhamnose, galacturonic acid, glucose, galactosel, xylose, arabinose and fucose).

### 3.5. Fourier Transform Infrared (FT-IR) Spectroscopy of MPP

MPP was mixed with spectroscopic grade potassium bromide (KBr) powder, ground and pressed into 1-mm pellets for FI-IR measurement. The FI-IR spectrum of MPP was determined using the Thermo antaris 2 spectrometer in the frequency range of 4000–400 cm^−1^.

### 3.6. Antitumor Activity in Vitro

#### 3.6.1. *In* *Vitro* Cancer Cell Line Cytotoxicity Assay

A549 non-small cell lung adenocarcinoma cell line was used to assay the antitumor activity of MPP. Cells were inoculated into 96-well plates at 1 × 10^4^ cells per well. RPMI-1640 containing antibiotics (100 μg/mL streptomycin and 100 U penicillin) and 10% FBS were used as the culture medium. After 4 h preincubation of the cells in a humidified 5% CO_2_ incubator at 37°C, MPP was added with final concentrations of 1, 10, 100, 500 and 1000 μg/mL, followed by further incubation for 24 h. Five mg/mL MTT solution dissolved in phosphate buffered saline (PBS) was added to each well by administering 10 μL per well. The cells were cultured for another 4 h, and then the culture medium was discarded. Finally, 100 μL dimethyl sulfoxide (DMSO) was added to each well, mixed, and measured at 570 nm.

#### 3.6.2. Assay of DNA Topoisomerase I (Topo I) Inhibitory Activity

Topo I inhibitory activity was assayed by relaxation of supercoiled pBR322 DNA according to the manufacturer’s instructions (Takara, Dalian, China). An amount of 0.5 μg supercoiled pBR322 DNA, 1 unit of topo I and different concentrations of MPP were incubated for 30 min at 37 °C in DNA Topo I buffer (10×: 350 mM Tris-HCl pH 8.0, 720 mM KCl, 50 mM MgCl_2_, 50 mM dithiothreitol (DTT), 50 mM spermidine) with 0.01% BSA, in a total volume of 20 μL. The effect of MPP on the plasmid DNA was assayed using the above method, but only 0.5 μg supercoiled pBR322 DNA and 200 μg/mL MPP were used for incubation at 37 °C for 30 min in DNA Topo I buffer to a total volume of 20 μL. MPP was diluted to 25, 50, 100, 200 and 400 μg/mL using H_2_O. The reaction was terminated by addition of 2 μL 10% sodium dodecyl sulfate (SDS) and 2 μL 6× loading dye solution. Samples were then electrophoresed in 1% agarose gel in Tris-acetate-ethylene diamine tetraacetic acid (EDTA) buffer for 40 min at 100 V. The gel was stained with Genview at room temperature and photographed with a UV transilluminator (G:Box, Syngene, Cambridge, UK). CPT was selected as reference drug. Topo I inhibitory activity was quantitated by determining the intensity of supercoiled and relaxed forms of plasmid DNA using the SynGene GeneTools imaging software.

### 3.7. Antioxidant Activity of MPP in Vitro

#### 3.7.1. DPPH Radical Scavenging Assay

The antioxidant activities of MPP were measured using the stable DPPH radical according to previous literature [26]. Briefly, 4 mL 0.004% methanol solution of DPPH was added to the sample solution at different concentrations (0.4–2.0 mg/mL). The mixture was shaken vigorously and left to stand for 30 min in darkness. Absorbance (A) was measured at 517 nm. The capacity to scavenge DPPH radical was calculated using the following equation.

Scavenging rate% = [(A_blank_ − A_sample_)/A_control_ × 100] (4)

where A_blank_ is the A of the control reaction (containing all reagents except the sample) and A_sample_ is the A in the presence of MPP. BHT was used as positive control.

#### 3.7.2. Hydroxyl Radical Scavenging Activity

Hydroxyl radical scavenging activity was measured by the Smiroff method with some modification [26]. Different concentrations (0.4, 0.8, 1.2, 1.6 and 2.0 mg/mL) of MPP were dissolved. One mL of sample solution was mixed with 1.0 mL 9.0 mM FeSO_4_, 1.0 mL 9.0 mM salicylic acid and ethanol solution, 1.0 mL 0.3% H_2_O_2_ and distilled water to 3 mL for 30 min at 37 °C. The hydroxyl radical was detected by monitoring at 510 nm. The hydroxyl radical scavenging effect was calculated as follows:

Scavenging rate % = (A_blank_ − A_sample_)/A_control_ × 100 (5)

where A_blank_ and A_sample_ represent the A of the blank control group and sample group under 510 nm, respectively. BHT was used as positive control.

#### 3.7.3. Superoxide Radical Scavenging Activity

Superoxide radical scavenging activity was determined according to the previous study [26]. Superoxide radical was generated by pyrogallic acid. MPP was dissolved in distilled water at 0.4, 0.8, 1.2, 1.6, and 2.0 mg/mL. The sample solution (1 mL) was mixed with 2 mL 0.05 mol/L Tris-HCl buffer (pH 8.2) and incubated at 25 °C in a water bath for 20 min. Then pyrogallic acid (0.4 mL, 5 mmol/L) was added, and the mixture was shaken rapidly at room temperature. The A value of the mixture was measured at 325 nm per 30 s against a blank. The scavenging ability to inhibit pyrogallol autoxidation was calculated using the equation:

Scavenging rate % = (S_blank_ − S_sample_)/S_control_ × 100 (6)

where S_sample_ represents the slope of the sample group, and S_blank_ is the slope of blank control group, where the decrease of S_sample_ indicates an increase in the restraining power. BHT was used as positive control.

#### 3.7.4. Reducing Power

The reducing power was determined using the previously described method with some modifications [27]. Varying concentration (0.4, 0.8, 1.2, 1.6 and 2.0 mg/mL) of MPP were placed in 2.5 mL 0.2 mol/L phosphate buffer (pH 6.6), to which 2.5 mL of potassium ferricyanide (1%) was added. The mixture was incubated at 50 °C for 20 min, followed by addition of 2.5 mL of trichloroacetic acid (10%). The mixture then was centrifuged at 6000× *g* for 10 min. The upper layer solution (2.5 mL) was mixed with 2.5 mL water and 1 mL FeCl_3_ (0.1%). Then, the A of the reaction mixture was read spectrophotometrically at 700 nm against a water blank. BHT was used as positive control.

#### 3.7.5. Total Antioxidant Activity 

The assay was based on the reduction of Mo (VI) to Mo (V) by the extract and subsequent formation of a green phosphate/Mo (V) complex at acid medium [28]. An amount of 0.3 mL of sample solution was combined with 3 mL reagent solution (0.6 mol/L sulfuric acid, 28 mmol/L sodium phosphate and 4 mmol/L ammonium molybdate). The tube was incubated at 95 °C for 90 min. After cooling the mixture to room temperature, the A value of the solution was measured at 695 nm against a blank. The antioxidant activity is expressed as the number of equivalents of ascorbic acid.

## 4. Conclusions

In the present study, we used RSM with a BBD to optimize the MPP extraction process and investigated its chemical composition and bioactivities. The optimal experimental yield (8.281% ± 0.387%) was achieved under the following extraction conditions: the ratio of water to raw material at 21.49, the extraction time of 1.44 h and the extraction temperature at 80.69 °C. Under these optimum conditions, the experimental extraction yield agreed closely with the predicted yield of 8.354%. In view of the practicality of operation, the extraction process is better when the ratio of water to material is at 20, extraction time of 1.5 h and extraction temperature at 80 °C, respectively. MPP is mainly composed of glucuronic acid, galacturonic acid, glucose, galactose and arabinose, and the molecular weight of its major fraction was estimated to be about 23.37 kD. MPP showed appreciable antitumor activity *in vitro*. Its inhibitory activity against topo I might be an effective way to achieve antitumor activity. The results of our antioxidant activity assay show that MPP possesses appreciable antioxidant activity and free radical scavenging activity* in vitro*. This study may provide a theoretical basis for systematic research, rational development and utilization of peppermint resources.
